# Neuroinflammation in Primary Cultures of the Rat Spinal Dorsal Horn Is Attenuated in the Presence of Adipose Tissue–Derived Medicinal Signalling Cells (AdMSCs) in a Co-cultivation Model

**DOI:** 10.1007/s12035-021-02601-9

**Published:** 2021-10-30

**Authors:** Stephan Leisengang, Laura B. Heilen, Michele C. Klymiuk, Franz Nürnberger, Daniela Ott, Kathrin Wolf-Hofmann, Rüdiger Gerstberger, Christoph Rummel, Martin J. Schmidt, Stefan Arnhold, Joachim Roth

**Affiliations:** 1 Institute of Veterinary Physiology and Biochemistry, Justus Liebig University Giessen, Frankfurter Strasse 100, 35392 Giessen, Germany; 2 Center for Mind, Brain and Behavior (CMBB), Philipps University Marburg & Justus Liebig University Giessen, Giessen, Germany; 3 Institute of Veterinary Anatomy, Histology and Embryology, Justus Liebig University Giessen, Frankfurter Strasse 98, 35392 Giessen, Germany; 4 Department of Veterinary Clinical Sciences, Small Animal Clinic, Justus Liebig University Giessen, Frankfurter Strasse 114, 35392 Giessen, Germany

**Keywords:** Superficial dorsal horn, Neuroinflammation, MSCs, Regenerative medicine, Immunomodulation, Glial activation

## Abstract

Neuroinflammation within the superficial dorsal horn (SDH) of the spinal cord induces inflammatory pain with symptoms of hyperalgesia and allodynia. Glial activation and production of inflammatory mediators (e.g. cytokines) is associated with modulation of nociceptive signalling. In this context, medicinal signalling cells, e.g. obtained from adipose tissue (AdMSCs), gained attention due to their capacity to modulate the inflammatory response in several diseases, e.g. spinal cord injury. We applied the recently established mixed neuroglial primary cell culture of the rat SDH to investigate effects of AdMSCs on the inflammatory response of SDH cells. Following establishment of a co-cultivation system, we performed specific bioassays for tumour necrosis factor alpha (TNFα) and interleukin (IL)-6, RT-qPCR and immunocytochemistry to detect changes in cytokine production and glial activation upon inflammatory stimulation with lipopolysaccharide (LPS). LPS-induced expression and release of pro-inflammatory cytokines (TNFα, IL-6) by SDH cells was significantly attenuated in the presence of AdMSCs. Further evidence for anti-inflammatory capacities of AdMSCs derived from a blunted LPS-induced TNFα/IL-10 expression ratio and suppressed nuclear translocation of the inflammatory transcription factor nuclear factor kappa B (NFκB) in SDH microglial cells. Expression of IL-10, transforming growth factor beta (TGF-β) and TNFα-stimulated gene-6 (TSG-6) was detected in AdMSCs, which are putative candidates for anti-inflammatory capacities of these cells. We present a novel co-cultivation system of AdMSCs with neuroglial primary cultures of the SDH to investigate immunomodulatory effects of AdMSCs at a cellular level.

## Introduction

The superficial dorsal horn (SDH) of the spinal cord is the first main site, where pain-related sensory information is integrated and modulated. Within the *laminae I* and *II*, central nerve endings of primary nociceptive neurons provide synaptic input to projection neurons, which forward the information to the brain, and interneurons that are able to regulate synaptic transmission within the SDH by the coordinated balance between inhibition and excitation [1, 2]. Inflammation and injury within structures involved in nociceptive processing result in symptoms of inflammatory and neuropathic pain, like hyperalgesia and allodynia [3]. Various animal studies in models of peripheral inflammation [4, 5], systemic inflammation [6, 7] and neuropathic pain [8] have highlighted the role of glial cells in spinal neuroinflammation modulating neuronal plasticity. Intrathecal application of endotoxin or cytokines was shown to produce hyperalgesia and allodynia [9–11]. It further leads to an increased expression of inflammatory mediators such as pro-inflammatory cytokines (e.g. TNFα, IL-6, IL-1β) and prostaglandins by glial cells [10, 12–14]. Cytokines are able to regulate neuronal activity by changing their responses to excitatory (e.g. glutamate) and inhibitory neurotransmitters [15–17]. Thus, inhibition of glial activation and production of inflammatory mediators are promising targets for novel pharmaceutical interventions [18, 19].

We recently established a model of inflammatory stimulation in a SDH primary cell culture using lipopolysaccharide (LPS) as inflammatory stimulus [20]. SDH primary cultures contain all cell types occurring within the SDH, like neurons (~ 44%), astrocytes (~ 13%), oligodendrocytes (~ 35%) and microglial cells (~ 9%). Therefore, SDH primary cultures are a useful tool to investigate glial activation and glia-neuron communication at a cellular level and share characteristics with classical in vivo or other in vitro models of inflammatory pain, such as microglial activation, release of cytokines and increased responsiveness of neurons to glutamate. Based on these attributes, we assume that primary SDH cultures can also be used to investigate possible anti-inflammatory effects of novel therapeutic approaches, like medicinal signalling cells (MSCs).

In the recent years, MSCs, also termed mesenchymal stem/stromal cells, gained attention due to their multipotent capacities in tissue regeneration and cell-based therapy. MSCs can readily be obtained from various tissues in adult animals, e.g. adipose tissue (AdMSCs) or bone marrow (BMSCs) [21], with MSCs of adipogenic origin showing higher rates of proliferation [22]. MSCs are able to differentiate into adipogenic, osteogenic and chondrogenic lineages in vitro, suggesting a potential to replace degenerated or injured tissue in a variety of diseases affecting the musculoskeletal system, not only in humans but also in animal patients [23–26]. Moreover, one main effect of their therapeutic potential seems to be the secretion of exosomes and soluble factors with anti-inflammatory or immunomodulatory capacities [27–29]. That is why Caplan [30] suggests to use the term ‘medicinal signalling cells’ instead of ‘mesenchymal stem cells’. Hitherto, it remains unclear which effect is determinative or if it is a combination of replacing injured tissue and the release of exosomes and soluble factors [31]. There is also some evidence that MSCs are capable to modulate neuroinflammation in states of inflammatory and neuropathic pain by inhibiting glial activation, cytokine expression and immune cell infiltration within the spinal cord, thereby reducing symptoms of mechanical allodynia and thermal hyperalgesia [32]. Several in vivo studies showed beneficial effects of MSC transplantation in animal models of neuropathic pain, like chronic constriction injury (CCI) [33, 34], diabetic neuropathy [35] and spinal cord injury (SCI) [36, 37].

The central goal of the present study was to establish a co-culture model of SDH primary cultures with AdMSCs and to use this approach to study effects on spinal neuroinflammation. We demonstrate modulatory effects of AdMSCs on the inflammatory response of SDH primary cultures upon LPS stimulation. Primary SDH cells cultured in the presence of AdMSCs show attenuated LPS-induced expression and release of pro-inflammatory cytokines (TNFα, IL-6) and a reduced nuclear translocation of the inflammatory transcription factor nuclear factor kappa B (NFκB) in microglial cells. We further show an increased LPS-induced expression of anti-inflammatory cytokines (IL-10) in AdMSCs. Overall, we suggest that the applied model of SDH/AdMSC co-cultures can be a useful tool to investigate immunomodulatory effects on spinal neuroinflammation at a cellular level.

## Methods

### Animals

Rats used for all experiments originated from an in-house breeding colony with parent animals from Charles River WIGA (Sulzfeld, Germany). All animals were kept at constantly monitored temperature (22 °C ± 1 °C) and relative humidity (50%) with ad libitum access to standard laboratory chow and water within a daylight cycle of 12 h of light from 7:00 AM to 7:00 PM. Animal care, breeding and experimental setup were performed according to the German Law on Animal Welfare, authorized by the Justus Liebig University Giessen (approval numbers GI 577_M and GI 580_M) and registered at the regional authority of Hessia, Germany.

### Preparation and Cultivation of SDH Primary Cultures

Primary cultures of the rat SDH were prepared as described previously [20]. In brief, 4- to 6-day-old Wistar rats were sacrificed by decapitation. The vertebral column was removed and cut into slices of 1 mm. The superficial dorsal horns of the spinal cord were extracted and enzymatically digested for 45 min using a mix containing collagenase (CLS II, 2.5 mg/ml; Biochrom GmbH, Berlin, Germany) and dispase II (5 mg/ml; Sigma-Aldrich Chemie GmbH, Taufkirchen, Germany) dissolved in oxygenated Hank’s Balanced Salt Solution (HBSS; w/o Ca^2+^ and Mg^2+^, Biochrom GmbH). After mechanical dissociation, the cell number was calculated using a Neubauer Improved Hemocytometer (NanoEntek, Seoul, South Korea) and cells were resuspended with a cell number of 120,000 cells/ml in a Neurobasal™ A (NBA) medium supplemented with 2% B27, penicillin (100 U/ml)/streptomycin (0.1 mg/ml) and 2 mM glutamine (all from Life Technologies GmbH, Darmstadt, Germany). Afterwards, a total volume of 350 µl of the cell suspension was transferred into special cultivation chambers composed of poly-l-lysine (0.1 mg/ml, Biochrom GmbH)–coated glass cover slips (Ø 13 mm, #2; Menzel, Braunschweig, Germany) bounded by flexiPERM© chambers (Micro12; Sarstedt AG & Co. KG, Nuembrecht, Germany). All cell cultures of one experiment originated from one cell suspension with the same cell number. Primary cell cultures were cultivated for 24 h at a temperature of 37 °C in a humidified atmosphere of 5% CO_2_ and 95% air.

### Preparation of AdMSCs

AdMSCs were isolated as previously described for the horse [38]. Briefly, 4- to 8-week-old Wistar rats were anesthetised with carbon dioxide and sacrificed by cervical dislocation. Abdominal fat was obtained from four distinct donors. Per donor, approximately 1 cm × 1 cm × 1 cm of fat tissue was extracted and cut into small pieces and the same volume of digestion medium containing collagenase (CLS I, 1 mg/ml, Biochrom GmbH), bovine serum albumin (10 mg/ml; Capricorn Scientific GmbH, Ebsdorfergrund, Germany) and phosphate-buffered saline (PBS; Life Technologies GmbH) was added. The digestion was implemented for 40–55 min in a rotator at 37 °C. The resulting homogeneous mixture was centrifuged at 240 g for 5 min. Subsequently, the detached cell pellet was filtered through a 70-µm cell strainer (Greiner Bio-One International GmbH, Kremsmünster, Austria). After a second centrifugation (240 g for 5 min), the supernatant was discarded and cells were resuspended in 1 ml cultivation medium containing Dulbecco’s Modified Eagle Medium low glucose (DMEM-LG; Life Technologies GmbH), penicillin (100 U/ml)/streptomycin (0.1 mg/ml) and 10% foetal calf serum (FCS; Bio & Sell GmbH, Feucht/Nürnberg, Germany). This suspension was plated in a 75-cm^2^ cell culture flask (Thermo Scientific, Waltham, MA, USA) containing 11 ml cultivation medium. Cultivation was performed at 37 °C in a humidified atmosphere of 5% CO_2_ and 95% air until a confluence of 80% was reached. The cells were passaged up to passage 3, which was used for the study.

### Characterization of AdMSCs

Since there are no standardized criteria for characterization of rat MSCs, we applied similar criteria, as described by Dominici et al. [39] for human MSCs. The expression of different cell surface markers, namely CD11b, CD29, CD45, CD73 and CD90, was detected by fluorescence-activated cell sorting (FACS) analysis. For this purpose, 100 µl of each cell suspension with 2 × 10^5^ cells was pipetted in wells of a 96-well microtitre plate with round bottom (Genaxxon bioscience, Ulm, Germany) and centrifuged at 400 g for 3 min. Afterwards, supernatants were discarded and the pellets were resuspended in washing buffer containing PBS, 0.01% NaN_3_ (Merck, Darmstadt, Deutschland) and 0.5% goat serum (Bio & Sell GmbH) and centrifuged as described above. Fifty microlitres of primary antibody (Table 1) or isotype control was pipetted in each well and incubated for 15 to 20 min. After incubation, two washing steps were performed as described above. Subsequently, for unconjugated primary antibodies, 50 µl of secondary antibody (Table 1) was pipetted in each well and incubated for 15 to 20 min. Finally, after two washing steps (washing buffer), the pellets were resuspended in 100 µl PBS and measured in the FACS BD Accuri™ C6 (Becton Dickinson, Heidelberg, Germany). The evaluation was performed with BD Accuri™ C6 software, version 1.0.264.21.

**Table 1:** Antibodies and isotype control for FACS analysis of AdMSCs

*Name*	*Manufacturer*	*Dilution*
**Conjugated primary antibodies**		
FITC mouse anti-rat CD45; clone: OX-1, Cat No.: 554877	BD Biosciences	1:200
APC mouse anti-rat CD11b; clone: WT.5, Cat No.: 562102	BD Bioscience	1:100
FITC hamster anti-rat CD29; clone: Ha2/5, Cat No.: 555005	BD Bioscience	1:400
PE mouse anti-rat CD90/mouse CD90.1; clone: OX-7, Cat No.: 551401	BD Bioscience	1:400
**Primary antibody**		
Purified mouse anti-rat CD73; clone: 5F/b9, Cat No.: 551123	BD Bioscience	1:20
**Isotype control**		
FITC mouse IgG1, κ isotype control; clone: MOPC-31C, Cat No: 550616	BD Biosciences	1:200
APC mouse IgA, κ isotype control; clone: M18-254, Cat No: 526140	BD Biosciences	1:100
FITC hamster IgM, λ1 isotype control; clone: G235-1, Cat No: 553960	BD Biosciences	1:400
Normal mouse IgG_1_-PE, Cat No.: sc-2866	Santa Cruz Biotechnology	1:400
Purified mouse IgG1, κ isotype control; clone: MOPC-31C, Cat No.: 557273	BD Biosciences	1:20
**Secondary polyclonal antibodies**		
m-IgGκ BP-PE, Cat No.: sc-516141	Santa Cruz Biotechnology	1:100

### Differentiation of AdMSCs

The multipotent differentiation of AdMSCs towards the three lineages (adipogenic, chondrogenic and osteogenic) is the most relevant criterion for AdMSC characterization [39]. Differentiation of AdMSCs was performed as previously described [40]. Adipogenic differentiation was implemented for 14 days in adipose differentiation medium. The differentiation medium contained Dulbecco’s Modified Eagle Medium high glucose (DMEM-HG; Life Technologies GmbH), penicillin (100 U/ml)/streptomycin (0.1 mg/ml), 5% FCS, dexamethasone (0.1 µM; Sigma-Aldrich, Steinheim, Germany), insulin-transferrin-selenium (ITS; 5 µg/ml, Sigma-Aldrich) and rosiglitazone (5 µM, Sigma-Aldrich). As control medium, DMEM-HG supplemented with penicillin (100 U/ml)/streptomycin (0.1 mg/ml) and 5% FCS was used. Samples were collected after 7 days and 14 days to perform Oil Red O staining. Briefly, after fixation in 4% formalin (Carl Roth, Karlsruhe, Germany), the cells were rinsed with 60% isopropanol (Carl Roth) and incubated for 30 min with Oil Red O solution consisting of three parts Oil Red O (Merck, Darmstadt, Germany) and two parts distilled water. Following incubation, cells were rinsed with distilled water and assessed microscopically. Finally, the Oil Red O was extracted with 100% ethanol and the optical density (OD) was measured with the microplate reader Sunrise™ (Tecan Group Ltd., Maennedorf, Switzerland) at 492 nm.

Chondrogenic differentiation was conducted for 21 days in a pellet culture. The chondrogenic differentiation medium consisted of DMEM-LG supplemented with penicillin (100 U/ml)/streptomycin (0.1 mg/ml), 5% FCS, ITS (10 µg/ml), ascorbic acid (0.17 mM, Sigma-Aldrich), sodium pyruvate (0.9 mM, Sigma-Aldrich), proline (0.35 mM, Sigma-Aldrich) and TGF-β1 (10 ng/ml, 100-21C; PeproTech, Hamburg, Germany). As control, cultivation medium with 5% FCS was used. After incubation for 21 days, the pellets were fixed in 4% formalin, embedded in paraffin and subjected for 30 min to Alcian blue staining containing Alcian blue (0.5 g, Sigma-Aldrich), glacial acetic acid (1 ml, Carl Roth) and distilled water (100 ml). Samples were counterstained with Nuclear fast red-aluminium sulfate solution composed of Nuclear fast red (0.1 g, Merck), aluminium sulfate (5 g, Merck) and water (100 ml) and were analysed microscopically.

For osteogenic differentiation, cells were cultured for 21 days in osteogenic differentiation medium. As control, cultivation medium with 5% FCS was used. Cultivation medium contained DMEM-LG supplemented with penicillin (100 U/ml)/streptomycin (0.1 mg/ml), 5% FCS, ascorbic acid (60 µM), dexamethasone (0.1 µM) and β-glycerophosphate (10 mM, Carl Roth). After cultivation, cells were fixed with 4% formalin, followed by incubation with 1% Alizarin Red S solution for 2 to 5 min. The staining solution contained Alizarin Red S (0.5 g, Carl Roth), 45 ml distilled water and 2.8 to 3.3% ammonia solution (Merck) for setting the pH value to 4.2. Subsequently, the samples were washed with distilled water and analysed microscopically.

### Co-cultivation of SDH Primary Cultures with AdMSC Cultures

For co-cultivation experiments, flexiPERM© chambers were removed and coverslips with SDH primary cultures were transferred into sterile tissue culture plates with 24 wells (VWR International GmbH, Darmstadt, Germany) under sterile conditions and supplied with 500 µl complete medium.

AdMSCs were pre-cultivated on TC inserts (pore size 0.4 µm, translucent, Sarstedt AG & Co. KG) developed for tissue culture plates with 24 wells. Thus, cells were unable to pass the membrane, while diffusion of soluble factors was possible. For pre-cultivation, 16 inserts per 24-well tissue culture plate were filled with 100 µl AdMSC cultivation medium, whereby 500 µl was added outside the inserts into the wells. In every insert, 2 × 10^4^ MSCs were seeded and incubated at 37 °C in a humidified atmosphere of 5% CO_2_ and 95% air. Following 24 h of incubation, the medium was changed to a mixture comprising both AdMSC cultivation medium and NBA medium, which is used for cultivation of SDH, in a ratio of 1:1. After further incubation for 24 h, the medium was changed to a 100% NBA medium, enabling AdMSC co-cultivation. This gradual change of medium leads to an improved habituation of MSCs to the complete medium, which was deduced from preliminary studies. Subsequent to incubation, the 24-well tissue culture plates with inlaying inserts were ready for co-cultivation.

**Fig. 1:**
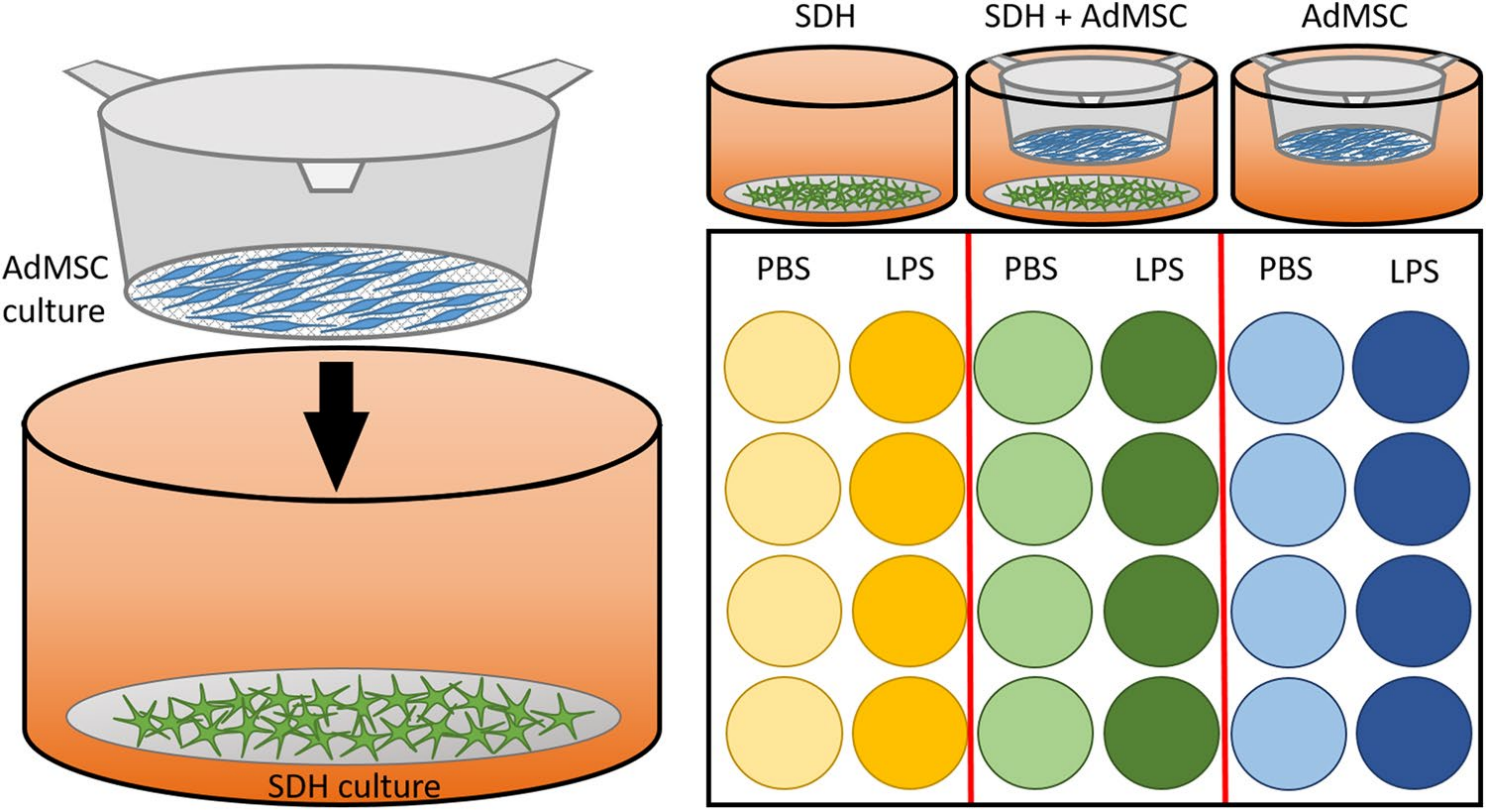
**Schematic illustration of the applied co-culture model of SDH primary cultures with AdMSC cultures.** After 1 day of cultivation, coverslips with SDH primary cultures were transferred into 24-well plates. AdMSCs were cultured on fine-pored inserts for 2 days, before they were applied for co-cultivation. Within the first group, SDH cells were cultured without AdMSCs (*SDH*, yellow), while the second group represented the co-cultivation group (*SDH* + *AdMSC*, green). In the third group, AdMSCs were cultured in the absence of SDH cells (*AdMSC*, blue). Eight wells per group were employed. Four of each group were used for inflammatory stimulation with LPS, while the remaining four wells were treated with PBS as a control. All supernatants were collected after 2 h of LPS stimulation. SDH and AdMSC cultures were separated and stored for further investigations (e.g. RT-qPCR, immunocytochemistry)

Eight wells of each plate were used to cultivate either only SDH cultures, or SDH cultures co-cultured with AdMSCs, or only inserts with AdMSC cultures (Fig. 1). After 24 h, all supernatants were removed and four wells per group were treated with NBA medium containing either bacterial LPS from *Escherichia coli* O111:B4 (10 µg/ml) or solvent in the same dilution (PBS, Sigma-Aldrich Chemie GmbH). After 2 h of stimulation with LPS or PBS, supernatants were collected and stored at − 20 °C for later cytokine measurements. SDH and AdMSC cultures were separated and immediately used for further investigations. SDH cultures were used for viability tests using trypan blue exclusion assay, fixation for subsequent immunocytochemistry or RNA extraction for later RT-qPCR. AdMSC cultures were used for dimethylthiazol-diphenyl tetrazolium bromide (MTT) colorimetric assay and RT-qPCR.

### Measurements of Cytokines TNFα and IL-6

We used two specific bioassays to detect the rather low amounts of TNFα and IL-6 released by cell cultures into the supernatants (for detailed description, see [41, 42]). For TNFα measurements, a bioassay based on the concentration-dependent cytotoxic effect of TNFα on the mouse fibrosarcoma cell line WEHI 164 subclone 13 was applied [43]. For calibration, we used the international murine TNFα standard (Code: 88/532; National Institute for Biological Standards and Control (NIBSC), South Mimms, UK). Briefly, biological samples in serial dilutions and standard in different concentrations were incubated for 24 h in sterile 96-well plates, which had previously been seeded with 50,000 WEHI cells treated with actinomycin D. Quantity of surviving cells was measured using the MTT colorimetric assay. Considering the dilution of samples, the TNFα assay detection limit proved to be 6.0 pg/ml. For determination of IL-6, we used a bioassay based on the concentration-dependent growth stimulation of IL-6 on a B9 hybridoma cell line [44]. Five throusand B9 cells were seeded in sterile 96-well plates, and biological samples in serial dilutions or standard in different concentrations (human IL-6 standard; Code: 89/548, NIBSC) were added. After an incubation time of 72 h, the number of cells was quantified using the MTT assay. The detection limit for the IL-6 assay proved to be 3.0 international units (IU) IL-6/ml.

### RT-qPCR

After stimulation experiments, all cells of four SDH primary cultures from the same group were pooled and lysed in 200 µl of lysis buffer (RA1 buffer, NucleoSpin© RNA XS kit; Macherey–Nagel, Düren, Germany). In one series of experiments, AdMSC cultures were separately lysed in 200 µl of lysis buffer (Lysis Solution, Product No. L8285, Sigma-Aldrich). For both cultures, RNA extraction was performed according to the manufacturer’s protocol using the NucleoSpin© RNA XS kit (Macherey–Nagel). RNA concentrations were adjusted to 5 ng/µl, and a total quantity of 40 ng was used for reverse transcription. In a first step, random hexamers (40 µM) and deoxynucleoside triphosphates (dNTPs; 10 µM) were added and RNA was denatured at a temperature of 65 °C for 10 min. For following reverse transcription, a mix of RT buffer, dithiothreitol (DTT; 0.1 M) and murine leukaemia virus reverse transcriptase (50 U; all: Applied Biosystems, Foster City, CA, USA) was applied at 37 °C for 60 min. In a last step of heating to 90 °C, the enzymes activity was stopped. Transcribed complementary DNA (cDNA) was stored at − 20 °C for later PCR experiments.

For relative quantification, the StepOnePlus Real-Time PCR System (Applied Biosystems) was applied. Using a mix of 5 µl TaqMan PCR Master Mix (Applied Biosystems), 3.5 µl sterile water and 0.5 µl primer with 1 µl cDNA, all probes were investigated in triplicates with the following protocol: polymerase activation (50 °C for 2 min), initial denaturation (95 °C for 10 min), 40 cycles of denaturation (95 °C for 15 s) and annealing/elongation (60 °C for 1 min). The following gene expression assays from Applied Biosystems were applied: IL-6, Rn01410330_m1; TNFα, Rn99999017_m1; IL-1β, Rn00580432-m1; IL-10, Rn99999012_m1; cyclooxygenase 2 (COX-2), Rn01483828_m1; TGF-β, Rn00572010_m1; and TSG-6, Rn01753871_m1.

To normalize cDNA quantities, β-actin (Rn00667869_m1; Applied Biosystems) was used as a housekeeping gene after comparing different possible housekeeping genes. For quantification, we used the 2^−(ΔΔCt)^ method. Results show the *x*-fold expression of a gene related to a control sample with the lowest expression, given a value of 1 for each gene.

### Immunocytochemistry

Immunocytochemistry was performed to investigate effects of co-cultivation of SDH primary cultures with AdMSC cultures on cell morphology and activation of the NFκB pathway. After fixation with 4% freshly prepared paraformaldehyde (PFA; Sigma-Aldrich Chemie GmbH) in PBS for 20 min, cells were washed three times in PBS and used for immunocytochemistry. A blocking solution containing 10% FCS (Capricorn Scientific GmbH) diluted in PBS-T containing 0.05% Triton X-100 (Sigma-Aldrich Chemie GmbH) was applied for 2 h to block unspecific binding sites. Cell cultures were incubated with primary monoclonal antibodies or polyclonal antisera (Table 2) diluted in blocking solution for 24 h at room temperature. After three times washing for 5 min with PBS-T, secondary antisera (Table 2) diluted in blocking solution were added for 2 h. Again, cells were washed three times and 2-(4-amidinophenyl)-1*H*-indole-6-carboxyamidine (DAPI; Invitrogen, Thermo Fisher Scientific, 1:10,000) was used to stain cellular nuclei. After washing for three times, coverslips were embedded using a glycerol/PBS solution (Citifluor Ltd., London, UK). Afterwards, immunoreactivity of SDH cultures was investigated using a fluorescence microscope (BX-50, Olympus Optical, Hamburg, Germany) equipped with appropriate filter sets and photographed and analysed using the MetaMorph imaging software (Molecular Devices, San Jose, USA). To examine the morphology and growth of neurons (MAP +), we measured the length of the longest process per neuron in two distinct experiments. In SDH microglial cells (CD68 +), the mean diameter was calculated, indicative for morphological changes upon activation. We further evaluated the immunoreactivity of nuclear NFκB signals by defining the nucleus as a region of interest and measuring the mean intensity of the respective channel within this area. All groups of one experiment were treated in the same immunocytochemical procedure and photographed and analysed under identical conditions.

**Table 2:** Antibodies and antisera for immunocytochemistry of SDH primary cultures

*Name*	*Manufacturer*	*Dilution*
**Primary monoclonal antibodies/polyclonal antisera**		
Mouse anti-MAP2 (2a + 2b), monoclonal IgG (M1406, Source: #22,190,325)	Sigma-Aldrich Chemie GmbH	1:600
Mouse anti-GFAP (2E1), monoclonal IgG (sc-33673, Lot: #K2116)	Santa Cruz Biotechnology	1:1000
Mouse anti-CNPase, monoclonal IgG (C-5922, Clone: 11-5B)	Sigma-Aldrich Chemie GmbH	1:1000
Mouse anti-rat CD68, clone ED1, monoclonal IgG (MCA341R, Lot: 148,922)	Bio-Rad Antibodies	1:1000
Rabbit anti-NFκB p65, polyclonal IgG (sc-109; Lot: #L247)	Santa Cruz Biotechnology	1:2000
Rabbit anti-STAT3 (C-20), polyclonal IgG (sc-482, Lot: #E1704)	Santa Cruz Biotechnology	1:6000
**Secondary polyclonal antisera**		
Alexa Fluor 488 donkey anti-mouse, polyclonal IgG (H + L) (A21202)	Thermo Fisher Scientific, Invitrogen	1:500
Cy3-conjugated AffiniPure donkey anti-rabbit, polyclonal IgG (H + L) (Code: 711–165-152, Lot: 148,356)	Jackson ImmunoResearch	1:2000

### MTT Assay

The MTT solution was prepared by dissolving MTT (5 mg, Carl Roth) in PBS (1 ml). Afterwards, MTT solution (10 µl) was added to the medium inside the inserts and incubated for 3 h at 37 °C in a humidified atmosphere of 5% CO_2_ and 95% air. The generated formazane was dissolved in acidified isopropanol (100 µl) for 10 min. To acidify isopropanol, 5% formic acid (Carl Roth) was used. Finally, the OD was measured with the microplate reader Sunrise™ at 570 nm.

### Evaluation and Statistics

To analyse effects of co-cultivation of AdMSC cultures with SDH primary cultures on the LPS-induced inflammatory response (cytokine release, gene expression, NFκB nuclear translocation), a two-way analysis of variance (ANOVA) was applied. Results of bioassays and nuclear translocation of transcription factor NFκB were obtained in three distinct preparations of SDH primary cultures with three distinct AdMSC donors, while gene expression was investigated in four distinct experiments. For evaluation of effective adipogenic differentiation, the Oil Red O staining data were analysed by paired *t* test. Thereby, the one tailed *p* value was applied. The software SigmaStat 4.0 (Systat Software, Inc., San José, CA, USA) and GraphPad Prism 5 and 7 (GraphPad Software, Inc., La Jolla, CA, USA) were used for analysis and creation of artwork. Results are illustrated as scatter plots with bars presenting the mean ± standard error of the mean (SEM).

## Results

### Evidence for AdMSC Stemness

**Fig. 2:**
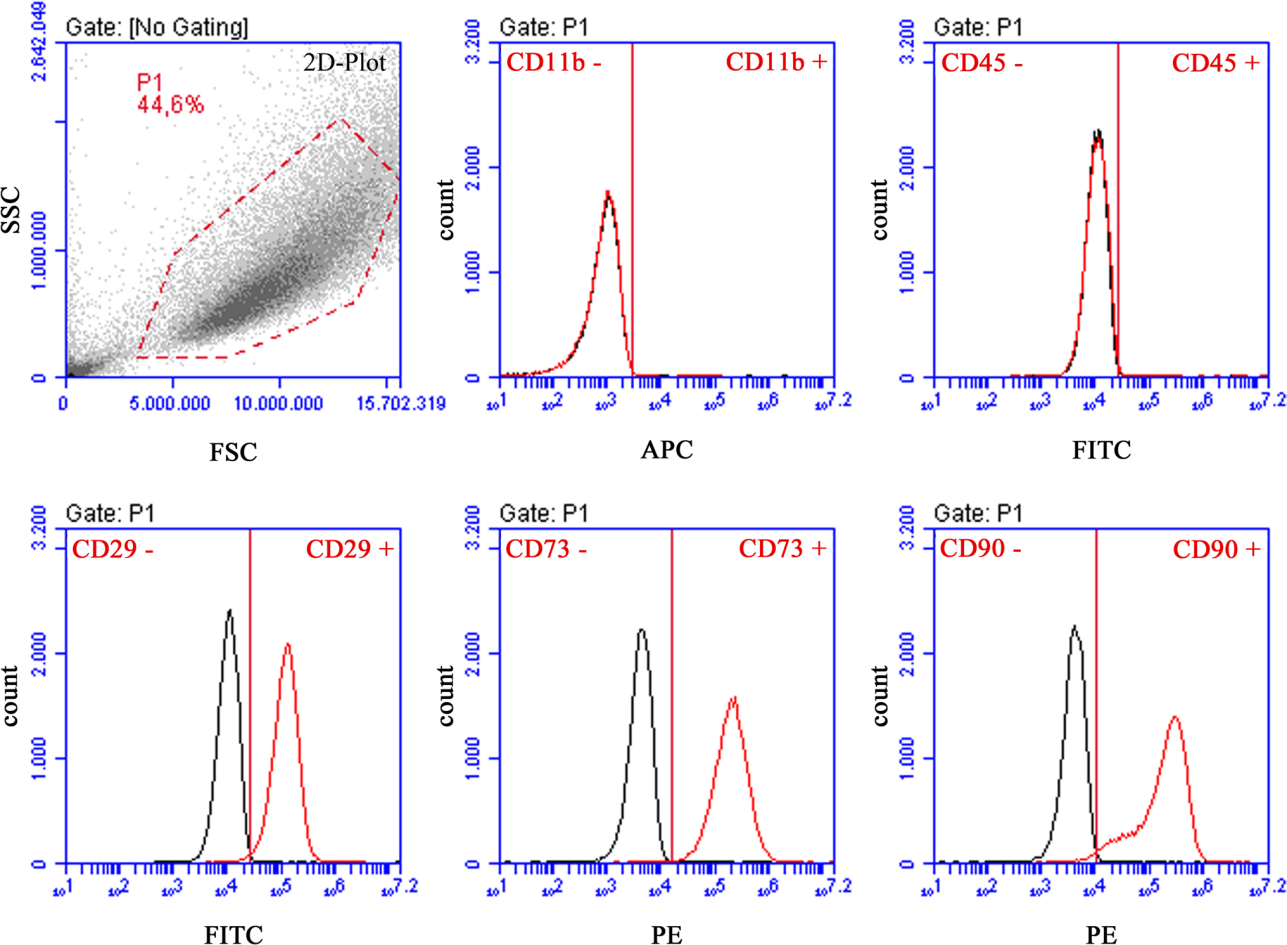
**Exemplary FACS analysis of AdMSCs.** FACS analysis was performed for the MSC-related cell surface markers CD11b, CD29, CD45, CD73 and CD90. Cell population is illustrated in a density plot to select the gate P1 for analysis. On the abscissa, the forward-scattered light (FSC, cell size) and, on the ordinate, the side-scattered light (SSC, cell granularity) are plotted. In the following graphs, black curves indicate isotype controls, while red curves represent experimental samples. Abscissae reflect counted cells. Ordinates show fluorescence intensities. AdMSCs demonstrated high expression of CD29, CD73 and CD90. Expression of haematopoietic stem cell markers CD11b and CD45 was negative. Exemplary illustration of one donor

Morphologically, AdMSCs were spindle-shaped and adhered to plastic surfaces. They showed a mean expression of over 90% for the positive markers CD29, CD73, and CD90. The negative markers CD45 and CD11b were expressed with a mean value of less than 1%. In Fig. 2, data from one donor are presented exemplarily. These findings show that AdMSCs possessed the required characteristics of cell surface marker expression. A detailed list of all values is listed in Table 3.

**Table 3:** Ratios of expressed cell surface markers for all donors of AdMSCs

Donor	Surface markers									
	CD 45		CD 11b		CD 29		CD 73		CD 90	
	+	−	+	−	+	−	+	−	+	−
1	1.1	98.9	0.9	99.1	78.3	21.7	99.6	0.4	88.8	11.2
2	0.8	99.2	0.7	99.3	99.0	1.0	99.9	0.1	98.8	1.2
3	0.61	99.39	0.29	99.71	98.98	1.02	99.69	0.31	98.42	1.58
4	1.0	99.0	0.9	99.1	99.0	1.0	99.9	0.1	92.8	7.2
Mean	0.88	99.12	0.7	99.3	93.82	6.18	99.77	0.23	94.71	5.29
SD	0.19		0.25		8.96		0.13		4.16	

**Fig. 3:**
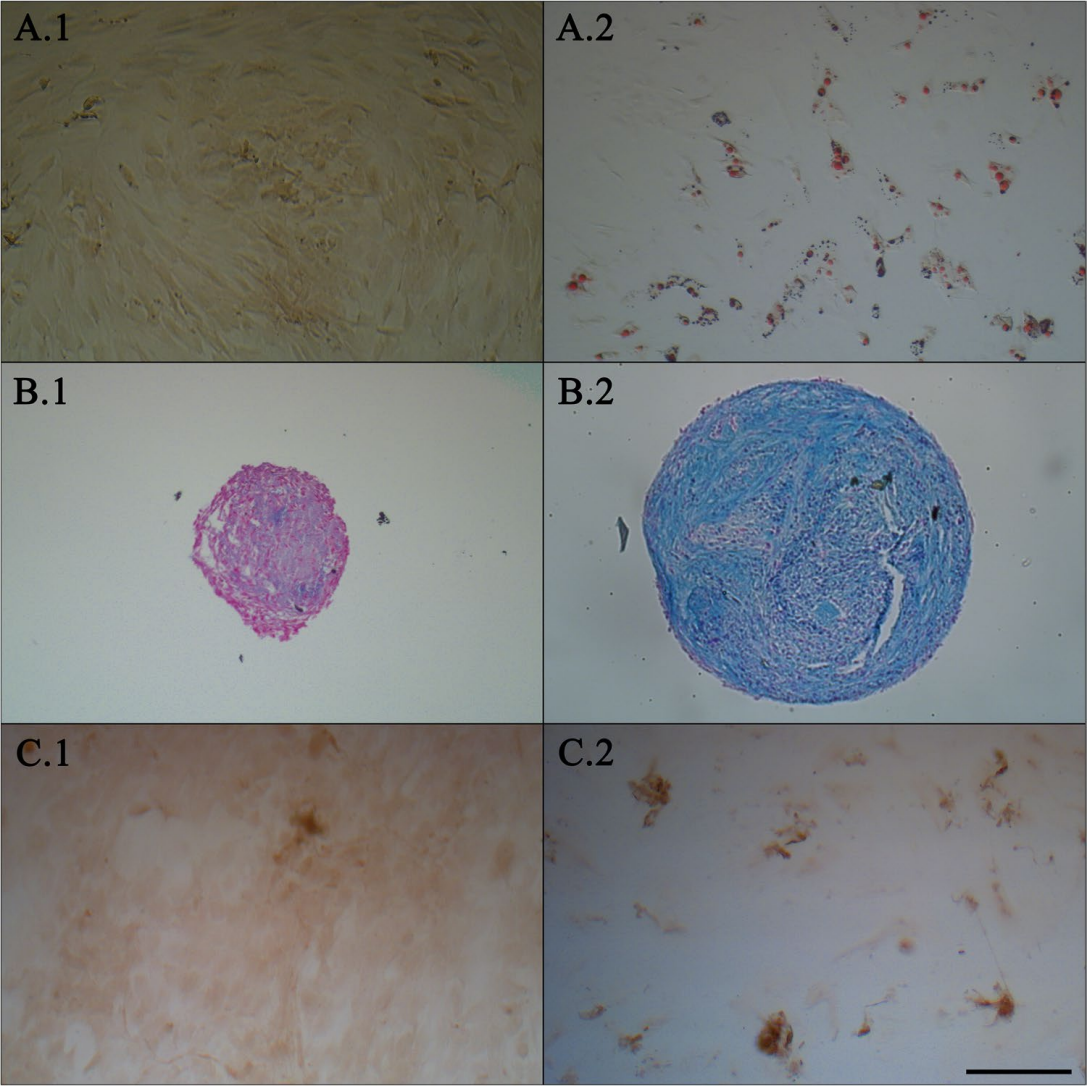
**Differentiation of AdMSCs towards adipogenic, chondrogenic and osteogenic lineages.** Oil Red O (**A**), Alcian blue (**B**) and Alizarin Red S (**C**) staining of AdMSCs grown in adipogenic (**A.2**), chondrogenic (**B.2**) and osteogenic (**C.2**) differentiation media and corresponding control media (**A.1**–**C.1**). Exemplary presentation of one donor. Scale bar in **C.2** represents 200 µm, applicable for all images

**Fig. 4:**
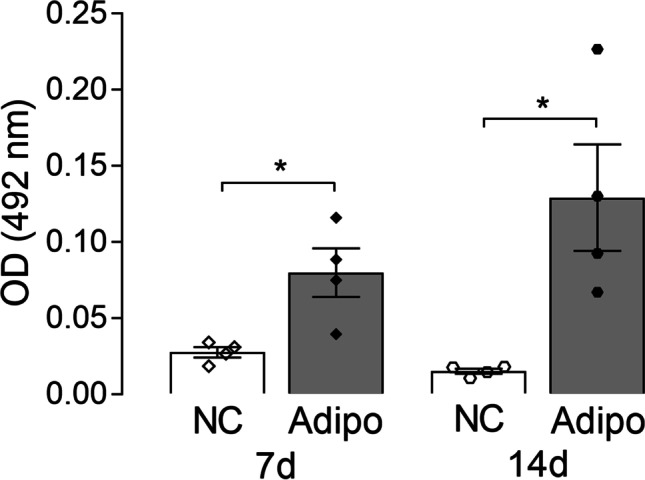
**OD measurement of Oil Red O staining for adipose-differentiated and undifferentiated AdMSCs.** OD measurement of Oil Red O after 7 days and 14 days of adipose differentiation (*Adipo*) resulted in a significant increase compared to the respective control (*NC*) incubated with control medium (7 days: *p = 0.036; 14 days: *p = 0.023, *t* test). Bars represent the mean ± SEM with symbols presenting the results of single experiments (*n* = 4)

As the most outstanding criterion, the differentiability was proven by a positive Oil Red O, Alcian blue and Alizarin Red S staining. Thereby, negative controls were unstained and, thus, remained undifferentiated (Fig. 3). Even the findings of OD measurement of Oil Red O staining for adipose-differentiated and undifferentiated AdMSCs on days 7 and 14 clearly demonstrated differentiability (Fig. 4).

### Effects of Co-cultivation with AdMSC on Viability, Cell Growth and Morphology of SDH Primary Cells

To investigate effects of AdMSCs on SDH primary cell cultures, we applied a model of co-cultivation of SDH cultures with AdMSC cultures growing on inserts with a fine-pored membrane allowing exchange of mediators but not cell migration. In one group, SDH cultures were cultivated without AdMSCs (*SDH*), while SDH cells of the second group grew in the presence of AdMSCs (*SDH* + *AdMSC*). Within the third group, AdMSC cultures were cultivated in inserts without SDH primary cultures (*AdMSC*) (Fig. 1). In a first step, we investigated effects of co-cultivation on cell viability and changes in cell growth and morphology of SDH primary cultures. We used a trypan blue exclusion test to compare cell viability between SDH cultures cultivated in the absence or presence of AdMSCs. Indeed, co-cultivation did not affect cell viability (data not shown).

Studies performed by other workgroups highlighted beneficial effects of MSCs on nerve regeneration and neurite outgrowth [45, 46]. We therefore investigated possible effects of co-cultivation with AdMSCs on growth and morphology of SDH neurons by means of immunocytochemistry. Calculating the length of the longest neuronal process per neuron, no significant effect on neuronal growth could be detected (SDH: PBS: 23.93 ± 0.72 µm, LPS: 22.68 ± 0.79 µm; SDH + AdMSC: PBS: 22.94 ± 0.75 µm, LPS: 23.15 ± 0.68 µm; all: mean ± SEM). Some authors describe changes in size and morphology of microglia due to activation or polarization into pro- or anti-inflammatory states [47–50]. Calculating the mean size of microglial cells, we did not detect quantifiable changes due to inflammatory stimulation or cultivation in the presence of AdMSCs (SDH: PBS: 16.22 µm ± 0.52, LPS: 15.84 µm ± 0.5; SDH + AdMSC: PBS: 17.34 µm ± 0.8, LPS: 16.36 µm ± 0.52).

### Effects of Co-cultivation with SDH Cells and LPS Stimulation on Viability, Cell Growth and Morphology of AdMSCs

**Fig. 5:**
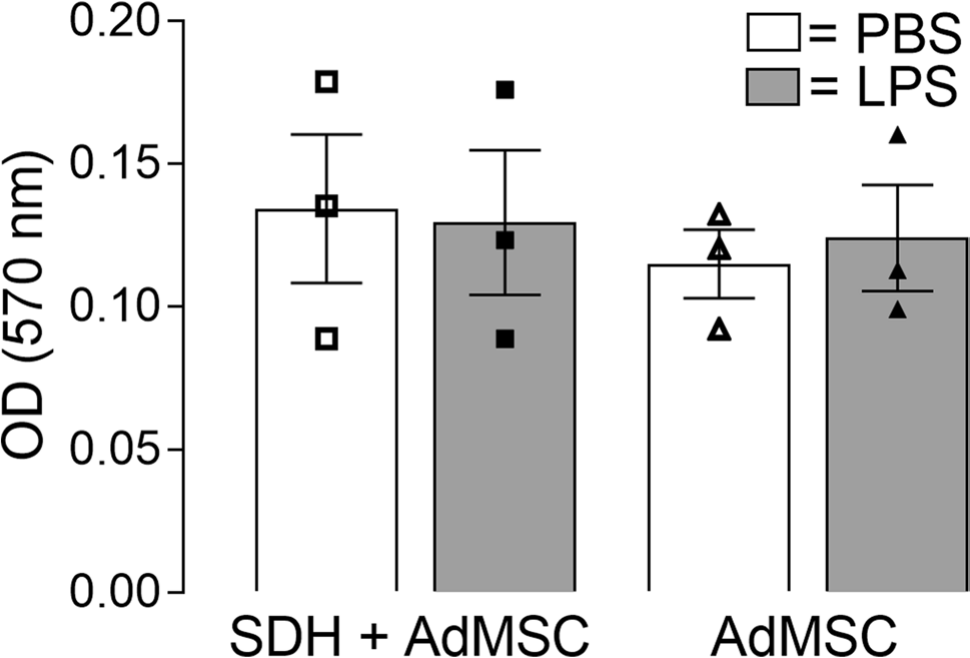
**MTT assay shows no significant effect on cell viability of AdMSCs.** MTT assay was implemented immediately after co-cultivation. No significant differences were detected between co-cultivation and solely cultivation of AdMSCs. Furthermore, no significant differences were determined for AdMSCs stimulated with LPS or PBS (control). Results are presented as mean ± SEM with symbols depicting results of single experiments (*n* = 3)

We used a MTT assay to compare the cell viability of AdMSCs cultured in the absence or presence of SDH. Furthermore, the effects of LPS stimulation on cell viability of AdMSCs were tested. Co-cultivation or LPS stimulation did not affect the cell viability of AdMSCs (Fig. 5). Thus, no statistical difference was detected for OD values of MTT neither for co-cultivation with SDH nor for LPS stimulation. Cell growth and morphology were evaluated microscopically. Thereby, no obvious differences could be found, when AdMSCs were cultivated in co-culture or stimulated with LPS (data not shown).

### Co-cultivation with AdMSCs Attenuates the Inflammatory Response of SDH Primary Cultures

We have recently shown that primary cell cultures are a useful tool for in vitro studies to investigate how inflammation, simulated by LPS stimulation, affects cells from the spinal dorsal horn, which are involved in nociceptive processing, e.g. increased expression and release of pro-inflammatory cytokines, activation of inflammatory transcription factors (NFκB, NF-IL6, STAT3) and modulation of neuronal responses to substance P and glutamate [20]. In the present study, we aimed to investigate whether AdMSCs are capable to modulate this inflammatory response.

**Fig. 6:**
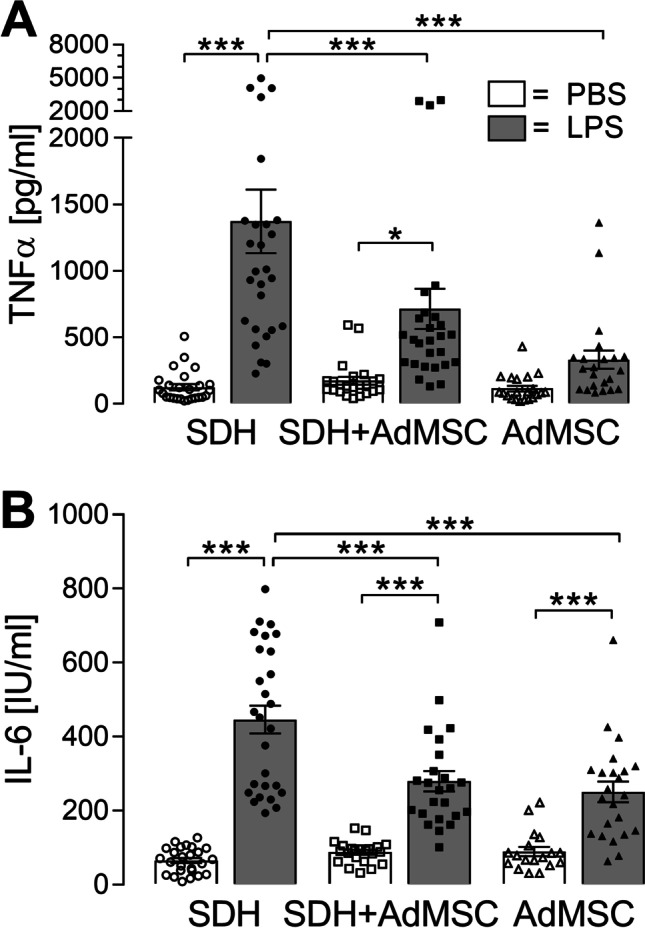
**LPS-induced release of pro-inflammatory cytokines TNFα and IL-6 is attenuated in the presence of AdMSCs.** Specific bioassays were applied to detect cytokines released into the supernatants after 2 h of stimulation. **A** Stimulation with LPS resulted in enhanced release of TNFα by SDH cells cultured in the absence (*SDH*: ***p < 0.001) or presence (*SDH* + *AdMSC*: *p < 0.05) of AdMSCs. The LPS-induced increase was significantly attenuated in the co-culture group compared to SDH cultures (***p < 0.001). AdMSCs (*AdMSC*) cultured in the absence of SDH cells did not show significant LPS-induced effects. **B** Release of IL-6 was significantly increased in all groups exposed to LPS (*all*: ***p < 0.001). However, LPS-induced release of IL-6 was significantly higher in SDH cells cultured in the absence of AdMSCs (*SDH, LPS*) compared to the co-culture group (*SDH* + *AdMSC, LPS*) and AdMSCs (*AdMSC, LPS*). Bars show the mean ± SEM with results of single experiments presented as symbols in the bars. A total number of 18–27 supernatants from three distinct preparations and three AdMSC donors were investigated

**Fig. 7:**
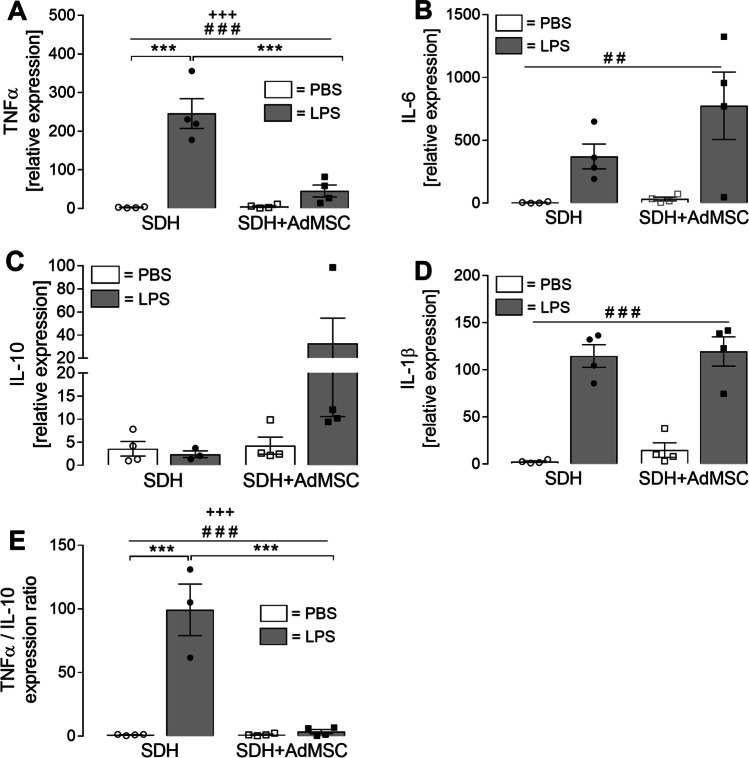
**Cytokine mRNA expression of SDH cells is modulated by co-cultivation with AdMSCs.** After 2 h of LPS stimulation, SDH cells were lysed, RNA was extracted and RT-qPCR was performed to analyse relative mRNA expression of pro- and anti-inflammatory cytokines. Expression of TNFα, IL-6 and IL-1β was increased upon treatment with LPS (**A**, **B**, **D**, treatment effect: ^###^*p* < 0.0001; ^##^*p* = 0.002). For TNFα (**A**), a co-culture effect was observed (^+++^*p* = 0.0004). LPS-induced expression of TNFα in SDH cultures is significantly elevated (***p < 0.001). This LPS-induced increase is significantly attenuated in the presence of AdMSCs (***p < 0.001), resulting in a non-significant elevation in the co-culture group. IL-10 expression (**C**) was not significantly modulated by LPS treatment or co-cultivation. Calculation of the TNFα/IL-10 ratio (**E**) can be used to assess a pro- or anti-inflammatory shift. Effects of LPS treatment (^###^*p* < 0.001) and co-cultivation (^+++^*p* < 0.001) were observed. LPS stimulation in SDH cultures shows a highly significant increase of TNFα/IL-10 ratio suggesting a pro-inflammatory shift (***p < 0.001). This increase is significantly attenuated in the co-cultivation group (***p < 0.001). Bars represent the mean ± SEM. Results originate from four distinct experiments and four AdMSC donors, indicated as single symbols

**Fig. 8:**
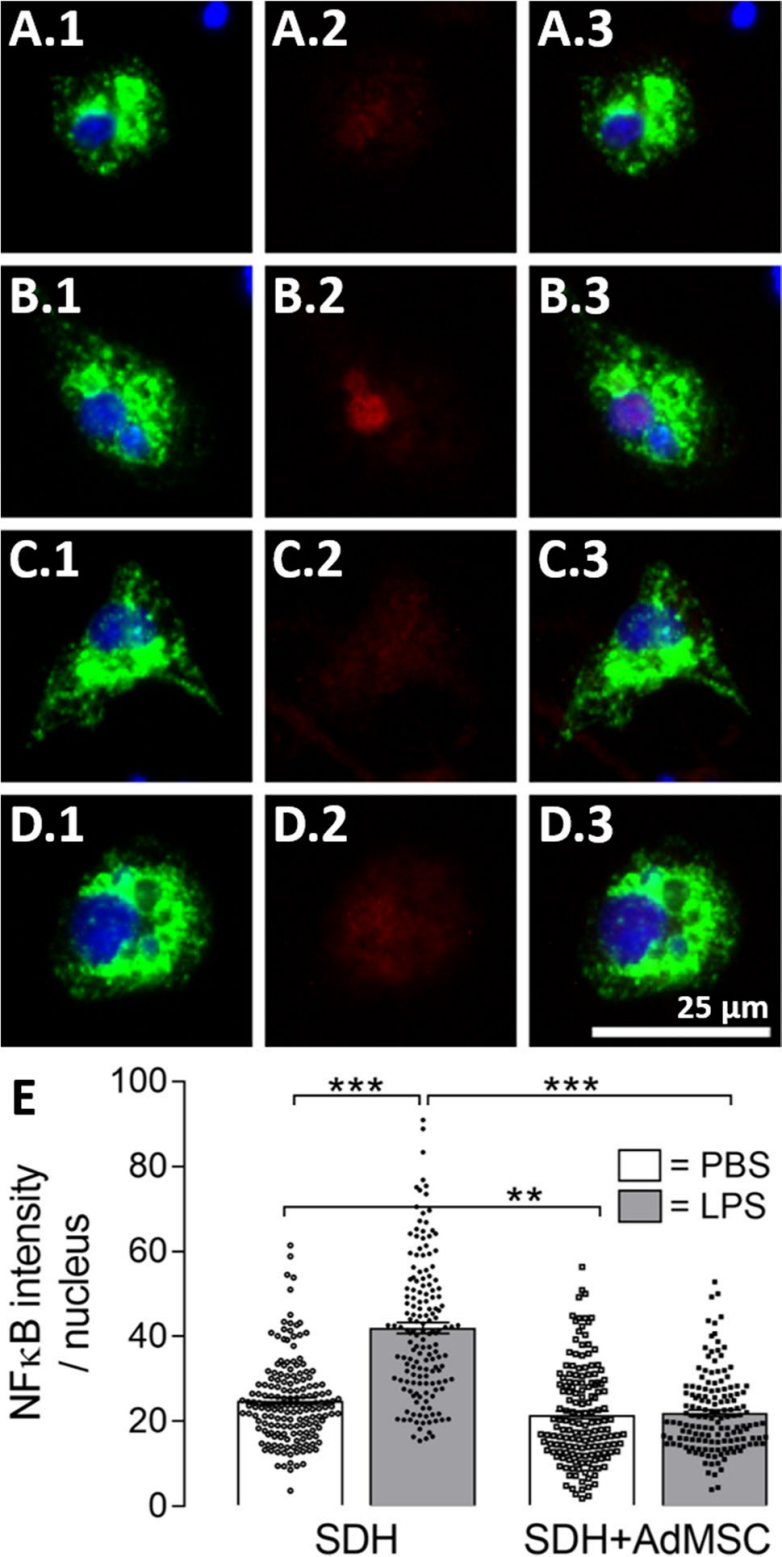
**AdMSCs suppress nuclear translocation of NFκB in spinal microglia upon LPS stimulation.** To investigate LPS-induced activation of inflammatory transcription factor NFκB in microglial cells, we performed immunocytochemistry using antibodies for NFκB (p65, red) and CD68 (green). Nuclei were stained with DAPI (blue). SDH microglial cells exposed to LPS showed increased nuclear NFκB signals (**B.1**–**B.3**) compared to PBS-treated cells (**A.1**–**A.3**). NFκB signals in microglial cells cultured in the presence of AdMSCs were barely visible (PBS: **C.1**–**C.3** and LPS: **D.1**–**D.3**). Selecting the nucleus as an area of interest, we were able to calculate the nuclear intensity of NFκB (**E**). Microglial cells stimulated with LPS for 2 h show significantly increased nuclear NFκB signals compared to the PBS control group (***p < 0.001). In the presence of AdMSCs, nuclear NFκB intensities were suppressed in LPS- and PBS-treated groups compared to SDH cultures (***p < 0.001; **p < 0.01). Scale bar represents 25 µm for all images. Bars presented in **E** show means ± SEM. Results of single microglial cells are illustrated as symbols and originate from three distinct experiments and three AdMSC donors (*SDH*: PBS: *n* = 170, LPS: *n* = 158; *SDH* + *AdMSC*: PBS: *n* = 156, LPS: *n* = 149)

**Fig. 9:**
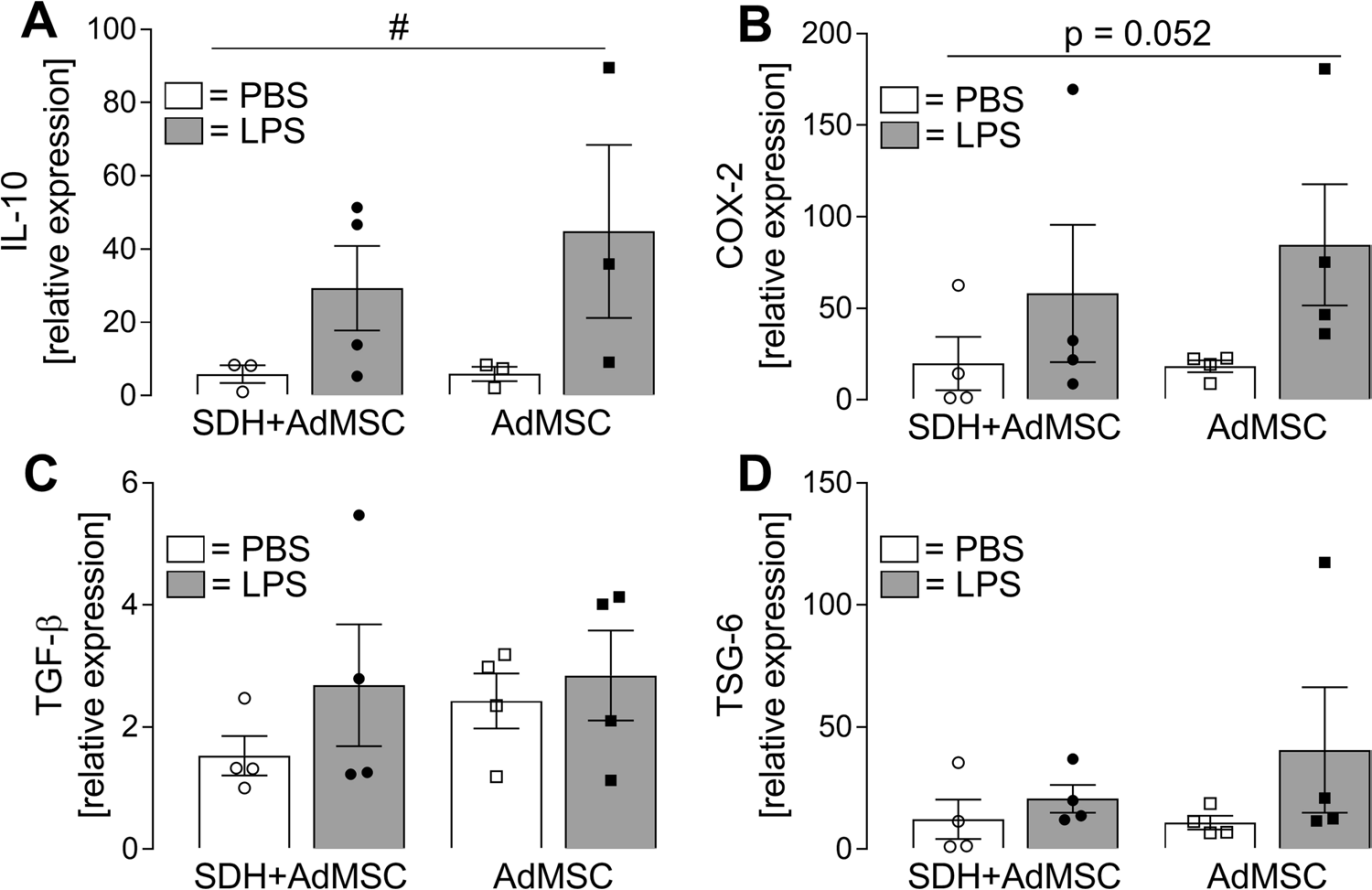
**Relative mRNA expression in AdMSCs after LPS stimulation cultured in the presence or absence of SDH primary cell cultures.** To investigate expression of putative immunomodulatory mediators, AdMSCs were lysed after LPS stimulation for RNA extraction. Relative expression of IL-10 (**A**), COX-2 (**B**), TGF-β (**C**) and TSG-6 (**D**) was determined by RT-qPCR. Stimulation with LPS resulted in a significant increase of IL-10 expression (treatment effect: ^#^*p* = 0.042), while an effect for COX-2 expression failed to obtain statistical significance (*p* = 0.052). No significant main effect was detectable for co-cultivation. TGF-β and TSG-6 expression levels were not significantly modulated by LPS or co-cultivation. Bars represent the mean ± SEM of four distinct experiments obtained from four AdMSC donors

After 24 h of co-cultivation, the nutrition media were exchanged by medium containing LPS (10 µg/ml) or solvent (PBS) in the same dilution. Supernatants were collected after 2 h of stimulation for measurements of cytokine release (Fig. 6), and SDH cells were used for RT-qPCR (Fig. 7) or immunocytochemistry (Fig. 8), while AdMSCs were used for viability tests and RT-qPCR (Fig. 9).

To detect the rather low amounts of TNFα released into the supernatants, a specific bioassay was performed (Fig. 6A). Stimulation with LPS resulted in a highly significant increase of TNFα release by SDH primary cultures (SDH: PBS: 125.88 ± 22.95 [*n* = 26] vs. LPS: 1373.07 ± 239.4 [*n* = 27]; *p* < 0.001). In co-cultures of SDH primary cells with AdMSCs, LPS stimulation leads to an increase of TNFα (SDH + AdMSC: PBS: 174.39 ± 29.33 [*n* = 23] vs. LPS: 715.07 ± 150.6 [*n* = 27]; *p* < 0.05). AdMSCs cultivated without SDH primary cells did not show significant changes in TNFα release due to LPS stimulation (AdMSC: PBS: 115.62 ± 21.27 [*n* = 21] vs. LPS: 333.05 ± 68.98 [*n* = 22]; *p* > 0.05). The LPS-induced TNFα release was significantly attenuated in the co-cultivation and AdMSC group compared to SDH cultures alone (SDH LPS: 1373.07 ± 239.4 vs. SDH + AdMSC LPS: 715.07 ± 150.6, *p* < 0.001; SDH LPS: 1373.07 ± 239.4 vs. AdMSC: 333.05 ± 68.98, *p* < 0.001). No significant changes were observed between PBS-treated groups.

Concentrations of IL-6 were measured by means of a specific IL-6 bioassay (Fig. 6B). Stimulation with LPS resulted in highly significant increases in IL-6 release into supernatants of all groups (SDH: PBS: 65.52 ± 6.91 [*n* = 25] vs. LPS: 446.37 ± 37.44 [*n* = 27]; SDH + AdMSC: PBS: 87.68 ± 6.5 [*n* = 22] vs. LPS: 279.46 ± 27.46 [*n* = 24]; AdMSC: PBS: 89.39 ± 12.48 [*n* = 18] vs. LPS: 250.74 ± 27.93 [*n* = 23]; *for all*: *p* < 0.001). However, the LPS-induced IL-6 release was significantly attenuated, when SDH cultures were cultivated in the presence of AdMSCs (SDH LPS vs. SDH + AdMSC LPS: *p* < 0.001). Interestingly, AdMSC cultures also showed an LPS-induced increase of IL-6 release, but significantly less compared to SDH cultures (SDH LPS vs. AdMSC LPS: *p* < 0.001).

The basal levels of TNFα and IL-6 within the PBS-treated co-cultivation group remained below the sum of concentrations of SDH cultures and AdMSCs cultured singularly, indicating an attenuated release of both cytokines upon co-cultivation, even under basal conditions.

To investigate the modulatory effects on messenger RNA (mRNA) expression of cytokines in SDH primary cultures, cells were lysed from coverslips after 2 h of stimulation and used for RNA extraction. After reverse transcription, the relative expression was determined by means of RT-qPCR (Fig. 7). Inflammatory stimulation with LPS resulted in increased expression of the pro-inflammatory cytokines TNFα, IL-6 and IL-1β (main effect *treatment*: TNFα: ^###^*p* < 0.0001; IL-6: ^##^*p* = 0.002; IL-1β: ^###^*p* < 0.0001). A main effect for *co-cultivation* was only determined for TNFα (^+++^*p* = 0.0004) with a significant interaction (*p* = 0.0004) (Fig. 7A). The LPS-induced increase of TNFα expression in SDH cultures was significantly attenuated in the model of co-cultivation with AdMSCs (SDH: PBS: 2.71 ± 0.64 vs. LPS: 245.87 ± 38.43, *p* < 0.001; SDH LPS vs. SDH + AdMSC LPS: 45.32 ± 15.42, *p* < 0.001). IL-10 is an anti-inflammatory cytokine involved in inflammation-resolving processes. In our experiments, only a tendency for an increased LPS-induced IL-10 expression was observed in the model of co-cultivation (SDH: PBS: 3.59 ± 1.59 vs. LPS: 2.39 ± 0.71; SDH + AdMSC: PBS: 4.27 ± 1.86 vs. LPS: 32.58 ± 22.02) (Fig. 7C). Calculating a TNFα/IL-10 ratio can reflect a shift from pro- to anti-inflammatory states or vice versa [51–53]. Indeed, LPS stimulation resulted in a significant treatment (^###^*p* < 0.0001) and co-culture effect (^+++^*p* < 0.0001) on the TNFα/IL-10 ratio. An LPS-induced increase, observed in SDH cultures (SDH: PBS: 1.09 ± 0.25 vs. LPS: 99.29 ± 20.25, *p* < 0.001), was blunted in SDH cultures cultivated in the presence of AdMSCs (SDH + AdMSC: PBS: 1.25 ± 0.43 vs. LPS: 3.66 ± 1.67, *p* > 0.05; SDH LPS vs. SDH + AdMSC LPS, ***p < 0.001) (Fig. 7E).

Microglia are known to be key modulators of inflammation within the spinal cord [54, 55], and their involvement in neuroinflammatory processes was also demonstrated in the model of SDH primary cultures, e.g. transcription factor activation and cytokine production [20]. Therefore, in the present study, we investigated effects of AdMSC co-cultivation on microglial activation by means of immunocytochemistry. Nuclear translocation of inflammatory transcription factors is indicative for microglial activation [56]. For immunocytochemical detection, we used antibodies against NFκB (p65 subunit) and CD68 (marker for macrophages and activated microglia) (Fig. 8). In SDH cultures, LPS stimulation resulted in an enhanced translocation of NFκB (red) into the area of the nucleus (blue) in microglial cells (green) (Fig. 8B.2 + B.3) compared to the PBS controls (Fig. 8A.2 + A.3). This marked LPS-induced nuclear translocation of NFκB was blunted in microglial cells co-cultured with AdMSCs (Fig. 8D vs. B). Selecting the nucleus as a region of interest, we measured the intensity of the NFκB signal in terms of grey levels (Fig. 8E). Statistical analysis showed a highly significant increase of nuclear NFκB signal in LPS-stimulated SDH primary cultures (SDH: PBS: 24.85 ± 0.75 vs. LPS: 42.02 ± 1.3, *p* < 0.001). Interestingly, not only LPS-induced increase in nuclear NFκB immunoreactivity was eliminated in co-cultivated cell cultures (SDH + AdMSC: PBS: 21.55 ± 0.92 vs. LPS: 21.95 ± 0.75, *p* > 0.05), but also basal nuclear NFκB immunoreactivity was reduced in PBS-treated groups (SDH PBS vs. SDH + AdMSC PBS: *p* < 0.01).

The presented data indicate anti-inflammatory effects of AdMSCs on SDH primary cultures in the model of co-cultivation. We, therefore, performed RT-qPCR of AdMSCs after LPS stimulation to detect mediators that are discussed to be involved in AdMSCs’ anti-inflammatory capacities, e.g. IL-10, TGF-β and TSG-6 (Fig. 9). Additionally, we examined the expression of COX-2, an enzyme involved in prostaglandin E_2_ (PGE_2_) synthesis. AdMSCs showed RNA expression of all four genes investigated. For IL-10, we detected a main effect for LPS treatment (^#^*p* = 0.04), which failed to reach statistical significance for COX-2 (*p* = 0.052). No modulatory effects on relative expression of TGF-β and TSG-6 upon LPS stimulation or co-cultivation were detected.

## Discussion

The results of our study can be summarized as follows: We demonstrate that the applied co-cultivation system can be used to investigate anti-inflammatory effects of AdMSCs on spinal dorsal horn cells. Expression and release of pro-inflammatory mediators (TNFα, IL-6) were significantly attenuated in the presence of AdMSCs. Nuclear translocation of the transcription factor NFκB in SDH microglial cells was blunted when co-cultured with AdMSCs. We could further show that AdMSCs express anti-inflammatory mediators (IL-10, TGF-β, TSG-6). We therefore suggest that the established co-cultivation model is a useful tool to study anti-inflammatory capacities of AdMSCs on spinal neuroinflammatory processes.

### Use of Primary Neuroglial Cell Cultures and Prospects of Models of Co-cultivation with MSCs

In previous studies, we have shown that primary neuroglial cell cultures are useful tools to investigate cellular responses upon inflammatory stimulation of various structures of the peripheral [57] and central [41, 42, 58–60] nervous systems. Preserving the specific composition of locally existing cell types (e.g. neurons and glial cells), such cultures can also be applied to investigate glia-neuron interactions at a cellular level. We recently established a primary culture of the rat SDH of the spinal cord, to study effects of inflammatory stimulation on cells of this structure [20]. Results of this study showed that SDH primary cultures share characteristics with experimental approaches under in vivo conditions investigating spinal neuroinflammation and emphasizing the role of glial cells in the context of inflammatory pain [10, 11]. In the present study, we aimed to investigate effects of AdMSCs on spinal dorsal horn cells in a model of co-cultivation. The use of inserts with a fine-pored, permeable membrane provides the opportunity to investigate effects of released soluble mediators by both cultures on each other without allowing direct contact [61, 62]. Stem cells of different origins have the capacity to stimulate neurite outgrowth and, therefore, support nerve regeneration in neurons of the peripheral nervous system [46, 63, 64]. This effect was improved by using MSCs differentiated into a Schwann cell phenotype for primary neurons from dorsal root ganglia in vitro [45] or in an in vivo model of sciatic nerve transection [65]. One study highlighted the role of released brain-derived neurotrophic factor (BDNF) by MSCs on axonal growth in cultured cells from the cortex and hippocampus of foetal rats [66]. However, in our experiments, cultivation of SDH primary cultures in the presence of AdMSCs did not affect neuronal growth or morphology. One reason for this discrepancy could be the cultivation period. Primary cell cultures are limited in the time of cultivation from 1 day [57, 67] to up to 6 days [41, 68], depending on the structure of interest. While glial cells, namely astrocytes, show increased differentiation and growth, the number of vital neurons decreases over time. To investigate effects of MSCs on neuronal regeneration, modifications in the cultivation procedure may be applicable. The age of the rats as well as the cultivation period or composition of nutrient media have significant impact on cell growth. Here, we adhered to the established protocol [20] to provide comparability of results between studies investigating effects of inflammatory stimulation and suggested anti-inflammatory mediators on SDH cultures.

### LPS-Induced Cytokine Release by SDH, AdMSC and Co-cultures

Neuroinflammation within the spinal dorsal horn, e.g. induced by intrathecal application of bacterial endotoxin (LPS), elicits allodynia and hyperalgesia [9, 10]. This LPS-induced effect is associated with an activation of glial cells and production of inflammatory mediators (e.g. cytokines, prostaglandins), which are able to modulate the synaptic transmission within the spinal dorsal horn [11, 69]. Recently, we reported that neurons of SDH primary cultures exposed to LPS showed altered responses to stimulation with substance P and glutamate in Ca^2+^ imaging experiments [20]. This effect was also related to an increased production of pro-inflammatory cytokines. In the present study, we first investigated LPS-induced release of the pro-inflammatory cytokines TNFα and IL-6 into the supernatants of SDH cultures (SDH), in co-cultures (SDH + AdMSC) and in AdMSC cultures (AdMSC) (Fig. 6). SDH primary cultures stimulated with LPS showed increased TNFα and IL-6 release into supernatants. In co-cultures, concentrations of both cytokines were significantly reduced upon LPS stimulation. These results are in line with in vivo studies investigating cytokine expression after SCI, suggesting immunomodulatory effects of stem cells [36, 37, 70]. In these studies, animals treated with stem cell transplantation after SCI showed not only reduced symptoms of pain hypersensitivity, but also reduced expression of pro-inflammatory cytokines (TNFα, IL-6, IL-1β). Manferdini et al. [61] showed similar effects of MSCs in cultured macrophages. Macrophages cultured in direct or indirect contact to AdMSCs showed reduced expression and release of pro-inflammatory mediators [61]. Thus, MSCs are capable to modulate cytokine expression within the spinal cord and in macrophage cultures. Interestingly, AdMSCs cultured in the absence of SDH cells also showed a significantly increased IL-6, but not TNFα release (Fig. 6). Indeed, MSCs express several Toll-like receptors (TLRs) [71, 72]. TLR4 is expressed by AdMSCs and recognizes bacterial LPS [73]. To this end, LPS-induced IL-6 release from AdMSC cultures in the present study is confirmed by previous results of others showing that TLR4 activation leads to modulation of the cytokine expression profile and an increased production of IL-6 by AdMSCs [74].

### Cytokine Expression of Cells from SDH Primary Cultures Is Modulated by AdMSC Co-cultivation

To further investigate the cytokine expression of SDH primary cell cultures cultured in the absence or presence of AdMSCs, RNA was extracted and RT-qPCR was performed (Fig. 7). We investigated pro-inflammatory cytokines (TNFα, IL-6, IL-1β), which have been shown to be upregulated in the spinal dorsal horn in several animal models of inflammatory and neuropathic pain [13]. For all three of these cytokines, we detected a highly significant main effect of LPS treatment. For TNFα, an additional effect of co-cultivation was observed. Notably, primary SDH cells cultured in the presence of AdMSCs showed significantly attenuated LPS-induced expression of TNFα mRNA. These results are in line with results of cytokine release (Fig. 6) and in vivo studies discussed in this context. Yet, we were not able to detect effects of co-cultivation on IL-6 and IL-1β mRNA expression. This may be due to time-dependent modulation of cytokine expression in the course of LPS-induced inflammation. Seo et al. [36] showed suppressed spinal IL-6 expression in MSC-treated rats after SCI in a very early phase (1 h after transplantation). However, later (1–3 days post transplantation), IL-6 expression was even increased in MSC-treated rats compared to the PBS-treated control group, supporting the idea of pro- and anti-inflammatory properties of IL-6 [75]. Other studies investigating effects of MSCs on cytokine expression in models of neuropathic pain show significantly attenuated IL-1β and IL-6 expression in later phases, e.g. after 24 h to several days post transplantation [33, 37, 76]. To evaluate effects of co-cultivation with AdMSCs on IL-1β expression, more experiments investigating additional time points after stimulation may be necessary. We further investigated mRNA expression of the anti-inflammatory cytokine IL-10. In SDH cultures cultured in the presence of AdMSCs, a tendency for an increased expression was observed compared to all other groups, however, not reaching statistical significance. Modulation of IL-10 expression by MSCs has previously been reported in in vivo models of inflammatory [77] and neuropathic [36] pain, but also in many other models of inflammation, e.g. sepsis [78], osteoarthritis [61] or traumatic brain injury [62]. In this context, the impact of macrophages has been discussed. Macrophages occur in different phenotypes. M1 macrophages, also called ‘classically activated’, can be activated by pathogen-associated molecular patterns (PAMPs), as LPS induces a pro-inflammatory response, e.g. TNFα production. M2, or ‘alternatively activated’, macrophages show an anti-inflammatory phenotype, e.g. IL-10 production, and are important to dampen the inflammatory response of an organism [79]. Depending on surrounding mediators, macrophages can switch between these phenotypes, called macrophage polarization. Such an effect has also been reported for spinal microglia, which are resident macrophages within the central nervous system [80, 81]. Considering TNFα as a pro-inflammatory marker and IL-10 as an important anti-inflammatory mediator, the TNFα/IL-10 ratio can provide some evidence for a shift in the balance of pro- and anti-inflammatory phenotypes [51, 53]. Our results show an eminent increase in the TNFα/IL-10 ratio in LPS-stimulated SDH cultures, representing a pro-inflammatory shift (Fig. 7E). This increase was blunted in the presence of AdMSCs. Thus, our results indicate effects of AdMSCs on microglial polarization.

### LPS-Induced Nuclear Translocation of NFκB Is Suppressed in SDH Microglial Cells Co-cultured with AdMSCs

To further investigate the activation of spinal microglial cells and its modulation by AdMSCs, we performed immunocytochemistry using antibodies for CD68 and NFκB (p65). CD68 is a protein of the lysosomal membrane of microglial cells and does not allow to discriminate between M1- and M2-polarized microglia [82, 83]. While some authors described changes in the morphology of microglia upon inflammatory stimulation [52–55], we were not able to identify quantifiable effects in the applied co-cultivation model by measuring the mean diameter in single microglial cells. Cultured microglia lose their physiological shape due to the dissociation process and recover during cultivation. Microglial morphology is affected by other cell types, as astrocytes, and the environment (nutrient media) [84, 85]. It is therefore a matter of debate, whether cell morphology is a useful readout to characterize microglial phenotypes in vitro. The canonical NFκB pathway is activated by several inflammatory stimuli, like PAMPs (e.g. LPS) via TLRs or cytokines (e.g. IL-1β, TNFα) via their specific receptors [86]. NFκB is expressed in most cells of an organism in its inactive form as heterodimer (e.g. p50/p65), bound to IκBα (inhibitor of NFκB). Activation is mediated via phosphorylation of IκB by IKKs (IκB kinase), resulting in IκB degradation. The remaining NFκB dimer translocates into the nucleus and binds to its specific promoter regions to regulate gene expression [87, 88]. Thus, nuclear translocation of NFκB is associated with inflammatory activation of a given cell [56]. Figure 8 shows increased nuclear signals of NFκB in spinal microglia upon stimulation with LPS. Co-cultivation with AdMSCs not only suppressed this LPS-induced translocation, but also reduced the basal immunoreactivity in control groups. Our results therefore indicate not only an impact of MSCs on LPS-induced nuclear translocation of NFκB in microglia, but also an overall reduced expression, at least for the p65 subunit, that was specifically detected by the applied antibody. NFκB activity depends on its dimerization. Five polypeptides can form 15 different NFκB dimers [88]. While the p50/p65 heterodimer mainly mediates transcription of pro-inflammatory genes (e.g. TNFα, IL-6), p50/p50 homodimers promote transcription of anti-inflammatory mediators such as IL-10 [89]. Yang et al. [76] observed reduced p65 protein in microglial cultures in the presence of BMSCs. Our results of reduced LPS-induced NFκB translocation by co-cultivation with AdMSCs, therefore, support a scenario of an anti-inflammatory shift in spinal microglia in the presence of these medicinal signalling cells (AdMSCs).

### Expression of Anti-inflammatory Mediators in MSCs

MSCs isolated from various tissues secrete immunoregulatory substances like IL-6, IL-10, TGF-β, TSG-6, PGE_2_, chemokines and further growth factors, which provide the opportunity to modulate the local inflammatory response by host immune cells [29, 90]. Therefore, they were also entitled ‘guardians of inflammation’ [28]. Due to the limited amount of extracted mRNA from MSC cultures, we had to decide for a selection of genes of interest for RT-qPCR analyses (IL-10, COX-2, TGF-β, TSG-6) (Fig. 9). In our model of co-cultivation, MSCs expressed all four investigated mediators, even under basal conditions. A significant effect of LPS stimulation was observed for IL-10 and was barely missed for COX-2 (*p* = 0.052), an enzyme involved in the synthesis of PGE_2_. LPS-induced IL-10 expression in AdMSCs has previously been observed [74, 91, 92], and IL-10 can reduce spinal neuroinflammation and improve motor function in animal models of SCI by altering macrophage phenotype [93]. Additionally, IL-10 prevents activation of NFκB and production of IL-6 in primary microglia cultured from the brain [94, 95]. However, IL-10 is expressed not only by MSCs, but also in resident macrophages and microglia in the presence of activated MSCs. MSCs activated by inflammatory mediators (e.g. LPS, TNFα, IL-6) show increased production of PGE_2_ [61, 78, 96]. Németh and colleagues [78] showed that PGE_2_ released by MSCs acts on EP_2_ and EP_4_ receptors to induce reprogramming of macrophages, thereby increasing IL-10 production. Moreover, in the context of macrophage/microglial polarization, involvement of TGF-β is also discussed [97, 98]. Treatment with BMSCs reduced symptoms of neuropathic pain in a model of CCI, inhibited glial activation and suppressed production of pro-inflammatory cytokines via TGF-β release, but not via IL-10 [92]. In our experiments, TGF-β was expressed by AdMSCs in all treatment groups, but was not significantly modulated by LPS stimulation or co-cultivation with SDH cultures. Similar results were obtained for TSG-6, another mediator released by MSCs that has been shown to exert anti-inflammatory capacities via macrophage/microglial polarization [76, 99]. In a mouse model of zymosan-induced peritonitis, TSG-6 suppressed NFκB signalling in resident macrophages and reduced production of pro-inflammatory mediators [100]. A very recent study from Yang and colleagues [76] demonstrated suppression of spinal neuroinflammation via TSG-6 secreted by BMSCs. In the same study, TSG-6 inhibited the NFκB signalling pathway in microglial cultures. Expression of anti-inflammatory mediators by AdMSCs, therefore, implicates distinct possibilities, by which AdMSCs might modulate glial activation and production of inflammatory mediators in SDH primary cultures. The presence of AdMSCs and the constitutive expression of immunomodulating mediators might affect SDH cells not only under inflammatory conditions (LPS), but also under basal conditions (PBS). However, further studies will be necessary to identify the precise mechanisms leading to the production and release of anti-inflammatory mediators by AdMSCs and how these substances act on SDH cells.

## Conclusions

### Putative Scenario of AdMSCs Anti-inflammatory Action on SDH Primary Cell Cultures in a Model of Co-cultivation

**Fig. 10:**
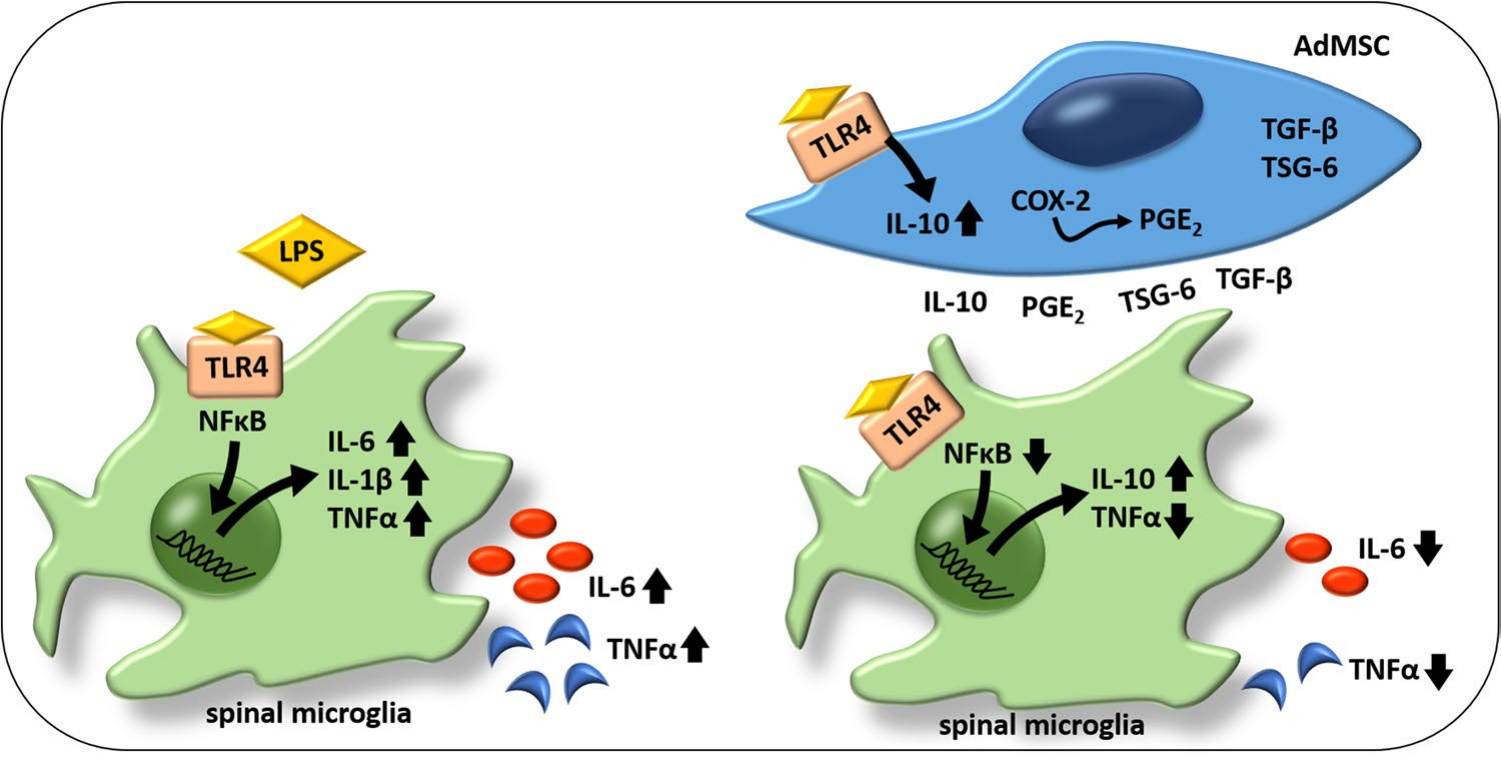
**Putative scenario of immunomodulatory effects of AdMSCs on SDH primary cells in the co-cultivation model.** SDH primary cultures exposed to LPS show increased nuclear translocation of NFκB in microglial cells and an increased expression and release of pro-inflammatory cytokines (TNFα, IL-6, IL-1β). AdMSCs express several mediators with immunoregulatory capacities (IL-10, TGF-β, TSG-6) and COX-2, an enzyme involved in PGE_2_ synthesis. In the presence of AdMSCs, SDH primary cell cultures show an attenuated inflammatory response upon LPS stimulation. Nuclear NFκB signals are significantly suppressed, and the release of pro-inflammatory cytokines (TNFα, IL-6) is attenuated. Calculating the TNFα/IL-10 ratio is indicative for a pro- or anti-inflammatory shift; AdMSCs inhibited the strong pro-inflammatory shift observed in the absence of AdMSCs. *COX-2* cyclooxygenase 2, *IL* interleukin, *LPS* lipopolysaccharide, *NFκB* nuclear factor kappa B, *PGE*_*2*_ prostaglandin E_2_, *TGF-β* transforming growth factor beta, *TLR4* Toll-like receptor 4, *TNFα* tumour necrosis factor alpha, *TSG-6* TNFα-stimulated gene-6 protein

Overall, we show that the introduced co-cultivation system of AdMSCs with SDH primary cultures represents a novel and reliable tool, to investigate immunomodulatory capacities of MSCs on spinal neuroinflammatory processes. The presented data provide evidence for the following putative scenario (Fig. 10): Spinal microglial cells exposed to LPS show increased nuclear translocation of the transcription factor NFκB and elevated expression and release of pro-inflammatory cytokines IL-6, IL-1β and TNFα. LPS stimulation in the presence of AdMSCs results in suppressed microglial NFκB activation and reduced production of TNFα and IL-6. The TNFα/IL-10 ratio indicates a diminished pro-inflammatory shift of SDH cells in the presence of AdMSCs. AdMSCs express mRNA for important anti-inflammatory mediators (IL-10, TGF-β, TSG-6) that have been shown to be involved in immunomodulatory effects of MSCs on spinal neuroinflammation, and for COX-2, an enzyme involved in PGE_2_ synthesis. PGE_2_, secreted by MSCs, can also induce macrophage/microglial polarization.

We suggest that the applied model of co-cultivation of SDH primary cultures with AdMSCs can be used to investigate immunomodulatory effects of MSCs on spinal neuroinflammatory processes and complement in vivo studies. Further experiments studying the impact of distinct mediators expressed by MSCs on SDH cells by stimulation with the respective substances or their respective inhibition in co-culture can be employed. In addition, it is possible to investigate effects of MSC-derived exosomes or MSC secretome–enriched media on SDH primary cultures. The introduced co-cultivation system can therefore contribute to a better understanding of immunomodulatory capacities of MSCs.

## Data Availability

The datasets used and/or analysed during the current study are available from the corresponding author on reasonable request.
